# Novel insights into genetic characteristics of *bla*_GES_-encoding plasmids from hospital sewage

**DOI:** 10.3389/fmicb.2023.1209195

**Published:** 2023-08-17

**Authors:** Yusuke Ota, Isaac Prah, Samiratu Mahazu, Yoshiaki Gu, Yoko Nukui, Ryuji Koike, Ryoichi Saito

**Affiliations:** ^1^Department of Molecular Microbiology and Immunology, Tokyo Medical and Dental University, Tokyo, Japan; ^2^Department of Parasitology and Tropical Medicine, Tokyo Medical and Dental University, Tokyo, Japan; ^3^Department of Infectious Diseases, Tokyo Medical and Dental University, Tokyo, Japan; ^4^Department of Infection Control and Laboratory Medicine, Kyoto Prefectural University of Medicine, Kyoto, Japan; ^5^Clinical Research Center, Tokyo Medical and Dental University Hospital, Tokyo, Japan

**Keywords:** GES, hospital sewage, plasmid, integron, toxin-antitoxin system

## Abstract

**Introduction:**

The prevalence of Guiana extended-spectrum (GES)-type carbapenemase producers is increasing worldwide, and hospital water environments are considered as potential reservoirs. However, the genetic features underlying this resistance are not yet fully understood. This study aimed to characterize *bla*_GES_-encoding plasmids from a single-hospital sewage sample in Japan.

**Methods:**

Carbapenemase producers were screened using carbapenemase-selective agar and polymerase chain reaction. Whole-genome sequencing analyzes were performed on the carbapenemase-producing isolates.

**Results:**

Eleven gram-negative bacteria (four *Enterobacter* spp., three *Klebsiella* spp., three *Aeromonas* spp., and one *Serratia* spp.) with *bla*_GES-24_ (*n* = 6), *bla*_GES-6_ (*n* = 4), and *bla*_GES-5_ (*n* = 1) were isolated from the sewage sample. Five *bla*_GES-24_ and a *bla*_GES-5_ were localized in IncP-6 plasmids, whereas three *bla*_GES-6_ plasmids were localized in IncC plasmids with IncF-like regions. The remaining *bla*_GES-6_ and *bla*_GES-24_ were, respectively, localized on IncFIB-containing plasmids with IncF-like regions and a plasmid with an IncW-like replication protein. The IncP-6 and IncW-like plasmids had a close genetic relationship with plasmids from Japan, whereas the IncC/IncF-like and IncFIB/IncF-like plasmids were closely related to those from the United States and Europe. All *bla*_GES_ genes were located on the class 1 integron cassette of the Tn*3* transposon-related region, and the IncC/IncF-like plasmid carried two copies of the integron cassette. Eight of the eleven *bla*_GES_-encoding plasmids contained toxin-antitoxin system genes.

**Discussion:**

The findings on the plasmids and the novel genetic content from a single wastewater sample extend our understanding regarding the diversity of resistance and the associated spread of *bla*_GES_, suggesting their high adaptability to hospital effluents. These findings highlight the need for the continuous monitoring of environmental GES-type carbapenemase producers to control their dissemination.

## 1. Introduction

The emergence of carbapenemase producers threatens the effective treatment of gram-negative bacterial infections, owing to their resistance to the most β-lactams, which are commonly used antibiotics, thus limiting therapeutic options (Gasink et al., 2009; Otter et al., 2017; Bonomo et al., 2018). Guiana extended-spectrum (GES)-type carbapenemase producers are prevalent in both clinical and environmental settings and are known to cause nosocomial outbreaks (Boyd et al., 2015; Naas et al., 2016; Yamasaki et al., 2017; Ellington et al., 2020; Literacka et al., 2020; Nakanishi et al., 2022; Yoo et al., 2023). The *bla*_GES_ genes are essentially linked to mobile genetic elements, which promote the spread of resistance genes to clinically relevant pathogenic bacteria and have been observed within integron gene cassettes on plasmids with diverse types of replicons (Naas et al., 2016). Although *bla*_GES_ genes have a high diffusion capacity in the medical environment (Nordmann and Poirel, 2014; Ellington et al., 2020), the genetic contexts of these resistance genes have not been extensively explored.

Hospital wastewater, which connects hospitals with the public health system, is a high-risk interface for the accumulation and effective dissemination of antimicrobial resistance (AMR) genes, increasing their burden on the environment, since the residual antibiotic levels of water may increase, allowing the selection for resistant microorganisms (Rodriguez-Mozaz et al., 2015; Bengtsson-Palme and Larsson, 2016; Mutuku et al., 2022). In addition, the aquatic environment serves as a potential hotspot for gene transfer and bacterial toxin-antitoxin systems, which are essential for the maintenance of these AMR genes, as well as for facilitating gene transfer among different bacterial species (Lee et al., 2015; An et al., 2018). Thus, the microbiological surveillance of hospital sewage is crucial to represent these risks and identify the route of dissemination of the resistant factors outside of hospital settings. Recently, the emergence of *bla*_GES_ has become an increasing environmental concern with potentially serious public health implications (Halat and Moubareck, 2020). However, the role of environmental transmission in GES-type carbapenemase producers has not been broadly researched, and the genetic features associated with environmental spread remain poorly understood.

This study aimed to investigate *bla*_GES_-harboring plasmids isolated from single-hospital sewage using whole-genome sequencing and demonstrate their genomic diversity. Our results provide further insights into the genetic context and impact on the evolution and expansion mechanism of *bla*_GES_ in hospital wastewater, elucidating the role of the healthcare water environment as a prospective reservoir of AMR.

## 2. Materials and methods

### 2.1. Wastewater collection and detection of carbapenemase producers

We collected untreated hospital sewage in a sterile bottle from one site of the Tokyo Medical and Dental University Hospital on May 30, 2022, and processed it within 2 h. The sample was concentrated 10×, and aliquots were plated on bromothymol blue (BTB) agar without antibiotics, BTB agar supplemented with ampicillin (32 μg/mL) and sulbactam (16 μg/mL), CHROMagar mSuperCARBA carbapenemase-selective agar (Kanto Chemical, Tokyo, Japan), and BTB agar containing colistin (4 μg/mL), which is reportedly associated with carbapenemase production (Bradford et al., 2015). The agar plates were incubated in ambient air conditions at 37°C. Bacterial colonies with distinct morphologies were subcultured on deoxycholate hydrogen sulfide lactose agar and gram-negative isolates were identified using 16S rRNA sequencing. The sequences were queried against the list of the Ribosomal Database Project,^1^ and the species were identified based on the closest relation (Cole et al., 2014). All gram-negative bacteria were transferred to CHROMagar mSuperCARBA agar for carbapenemase screening, and the selected colonies were tested for the presence of carbapenemase-encoding genes (*bla*_IMP_, *bla*_VIM_, *bla*_KPC_, *bla*_GES_, *bla*_OXA-48-like_, and *bla*_NDM_) using standard polymerase chain reaction (PCR), as described previously (Dallenne et al., 2010; Wachino et al., 2011).

### 2.2. Antimicrobial susceptibility testing

The minimum inhibitory concentrations (MICs) of antibiotics were determined using the broth microdilution method on a commercial Dry Plate (Eiken Chemical, Tokyo, Japan). The tested antibiotics were as follows: piperacillin, piperacillin/tazobactam, cefoperazone/sulbactam, cefozopran, ceftriaxone, ceftazidime, cefepime, aztreonam, imipenem, meropenem, doripenem, gentamicin, amikacin, tobramycin, minocycline, levofloxacin, ciprofloxacin, sulfamethoxazole/trimethoprim, and colistin. The results were analyzed using an image analyzer IA01 MIC Pro (Eiken Chemical). The breakpoints for antimicrobial susceptibility testing results were based on the CLSI documents M100-Ed32 (*Enterobacterales*) and M45-Ed3 (*Aeromonas* spp.). *Escherichia coli* ATCC 25922 was used as a quality control strain.

### 2.3. Whole-genome sequencing and analysis

Bacterial DNA was extracted from the resulting colonies of each isolate using a NucleoBond HMW DNA Kit (Macherey-Nagel, Düren, Germany). Library preparation was conducted using the Nextera DNA Flex Library Prep Kit (Illumina, San Diego, CA, United states) for Illumina short reads, and paired-end whole-genome sequencing was performed using the Illumina MiniSeq platform, following the manufacturer’s protocol. A DNA library for long reads was prepared using a Ligation Sequencing Kit and then sequenced using a Nanopore MinION sequencer and R9.4.1 flow cell (Oxford Nanopore Technologies), according to the manufacturer’s instructions. Filtering and trimming of raw short reads were performed using Fastp v0.22.0 (An et al., 2018) and long reads were quality-filtered using Porechop v0.2.4 (Wick et al., 2017a) and NanoFilt v0.2.1 (De Coster et al., 2018). The filtered reads were *de novo* hybrids assembled using Unicycler v0.5.0 (Wick et al., 2017b). Circularization of the assembled contigs was confirmed using Bandage v.0.8.1 (Wick et al., 2015). Assembled contigs were annotated using Rapid Annotation using Subsystem Technology v2.0 (Aziz et al., 2008). Bacterial species were identified based on the average nucleotide identity values using the GTDB Toolkit Classify v1.6.0 (Chaumeil et al., 2020). Sequence type (ST) was determined using multi-locus sequence typing (MLST) v2.0 tool^2^ and was assigned to isolates using the MLST database^3^ (Larsen et al., 2012). The STs of *Klebsiella variicola* were determined using a previously reported database^4^ (Barrios-Camacho et al., 2019). Acquired AMR genes were detected using ResFinder v4.1 (Zankari et al., 2012) and Basic Local Alignment Search Tool (BLAST) (Altschul et al., 1990). PlasmidFinder v2.0 was used to identify the incompatibility (Inc) type of the plasmids (Carattoli et al., 2014). Integrons were detected using IntegronFinder v2.0 (Neron et al., 2022). ISfinder was used to identify transposon and insertion sequences (Siguier et al., 2006). The BLAST Ring Image Generator v0.95 was used to align assembled reads containing *bla*_GES_ with closely related plasmids available in the GenBank genome database (Alikhan et al., 2011). EasyFig v2.2.2 was used for a linear comparison of the genetic context of *bla*_GES_ (Sullivan et al., 2011). A maximum likelihood phylogenetic tree of *bla*_GES_-encoding plasmids and the high-homology plasmid from GenBank was constructed using the MEGA X software (Kumar et al., 2018).

### 2.4. Transferability of the *bla*_GES_-encoding plasmids

We examined the transferability of plasmids carrying *bla*_GES_ using bacterial conjugation and transformation methods as described previously, with some modifications (Ota et al., 2022). Based on the Inc. type and the phylogenetic tree of *bla*_GES_-encoding plasmids, *Enterobacter kobei* AS2, *K. variicola* AS3, *K. variicola* AS10, and *Aeromonas hydrophila* CL1 were selected as donor strains. Bacterial conjugation was performed using the agar mating method with sodium azide-resistant *Escherichia coli* J53 and rifampicin-resistant *Escherichia coli* C600 as recipient strains. Exponential-phase Luria-Bertani (LB) broth cultures of donor and recipient strains were mixed in equal proportions and incubated on LB agar plates at 37°C. The conjugation mixture was plated on BTB agar plates with 2 μg/mL meropenem or 2 μg/mL cefotaxime plus 50 μg/mL rifampicin for *Escherichia coli* C600 recipients or 100 μg/mL sodium azide for *Escherichia coli* J53 recipients, to select for potential transconjugants. For transformation, plasmid DNA was extracted from the donor strains using the NucleoBond Xtra Midi Kit (Takara Bio, Shiga, Japan) and then electroporated into *Escherichia coli* HST08 Premium Electro-Cells (Takara Bio). The probable transformants were selected on LB agar plates supplemented with either 2 μg/mL meropenem or 2 μg/mL cefotaxime. The *bla*_GES_ transconjugants and transformants were screened using PCR and antimicrobial susceptibility testing (Dallenne et al., 2010). To confirm the localization of *bla*_GES_, short-read whole-genome sequencing was conducted using a MiniSeq sequencing platform, and plasmid-derived reads were assembled using PlasmidSPAdes (Galaxy Version 3.15.4+ galaxy2) (Giardine et al., 2005; Antipov et al., 2016). Conjugation frequency was determined as the number of transconjugants obtained per recipient cell.

## 3. Results

### 3.1. Isolation of GES-type carbapenemase producers from hospital sewage

A total of 38 gram-negative bacteria were recovered from hospital sewage and identified using 16S rRNA gene sequencing (Supplementary Table S1). Among them, 11 isolates (four *Enterobacter* spp., three *Klebsiella* spp., three *Aeromonas* spp., and one *Serratia* spp.) were identified as GES-type-β-lactamase producers using standard PCR, but no carbapenemase genes other than *bla*_GES_ were detected. Hence, these isolates were used for short- and long-read sequencing (Table 1). Each isolate was classified as a phylogenetically distinct strain with an average nucleotide identity value of more than 95.0%, based on whole-genome species identification and MLST. The *bla*_GES_ genes of all 11 isolates were detected on circular plasmids and identified as *bla*_GES-24_ (*n* = 6), *bla*_GES-6_ (*n* = 4), and *bla*_GES-5_ (*n* = 1). The *bla*_GES_-encoding plasmids AS12 and CL1 also contained the OXA-17 and PAC-1 β-lactamase genes, respectively.

**Table 1:** Bacterial species, sequence type, and genome characteristics of *bla*_GES_-encoding plasmids.

Strain	Species	Sequence type	*bla*_GES_-containing plasmid				
			Component	Length (bp)	Circular/Linear	Inc type	Antibiotic resistance gene
AS1	*Enterobacter soli*	ND	AS1_contig4	17,185	Circular	IncP-6	*aadA5*, *sul1*, *bla*_GES-24_, *catB*, *qacE*
AS2	*Enterobacter kobei*	ST32	AS2_contig2	142,147	Circular	IncFIB, IncF-like	*sul1*, *bla*_GES-6_, *qacE*
AS3	*Klebsiella variicola*	No match	AS3_contig8	30,777	Circular	IncP-6	*aac(6')-Il*, *aac(6')-31*, *sul1*, *bla*_GES-24_, *catB*, *qacE*
AS4	*Enterobacter roggenkampii*	ND	AS4_contig2	150,760	Circular	IncC, IncF-like	*sul1*, *bla*_GES-6_, *qacE*
AS8	*Enterobacter kobei*	ST910	AS8_contig4	15,748	Circular	IncP-6	*aac(6')-Ia*, *sul1*, *bla*_GES-5_, *qacE*
AS10	*Klebsiella variicola*	ST289	AS10_contig3	170,503	Circular	IncC, IncF-like	*sul1*, *bla*_GES-6_, *qacE* (2 copies each)
AS12	*Aeromonas hydrophila*	ST721	AS12_contig5	33,677	Circular	IncP-6	*aac(6')-Ib-cr*, *aac(6')-Ib3*, *sul1*, *bla*_OXA-17_, *bla*_GES-24_, *qacE*
CL1	*Aeromonas hydrophila*	ST2205	CL1_contig4	37,107	Circular	IncW-like	*aac(6')-Il*, *aac(6')-31*, *aadA1*, *sul1*, *bla*_GES-24_, *bla*_PAC-1_, *catB*, *qacE*
CA1	*Klebsiella quasipneumoniae*	ND	CA1_contig5	30,777	Circular	IncP-6	*aac(6')-Il*, *aac(6')-31*, *sul1*, *bla*_GES-24_, *catB*, *qacE*
CA4	*Aeromonas dhakensis*	No match	CA4_contig4	159,779	Circular	IncC, IncF-like	*sul1*, *bla*_GES-6_, *qacE*
CA6	*Serratia marcescens*	ND	CA6_contig7	30,777	Circular	IncP-6	*aac(6')-Il*, *aac(6')-31*, *sul1*, *bla*_GES-24_, *catB*, *qacE*

ND: not determined.

### 3.2. Structural characterization of *bla*_GES_-harboring plasmids

**Figure 1:**
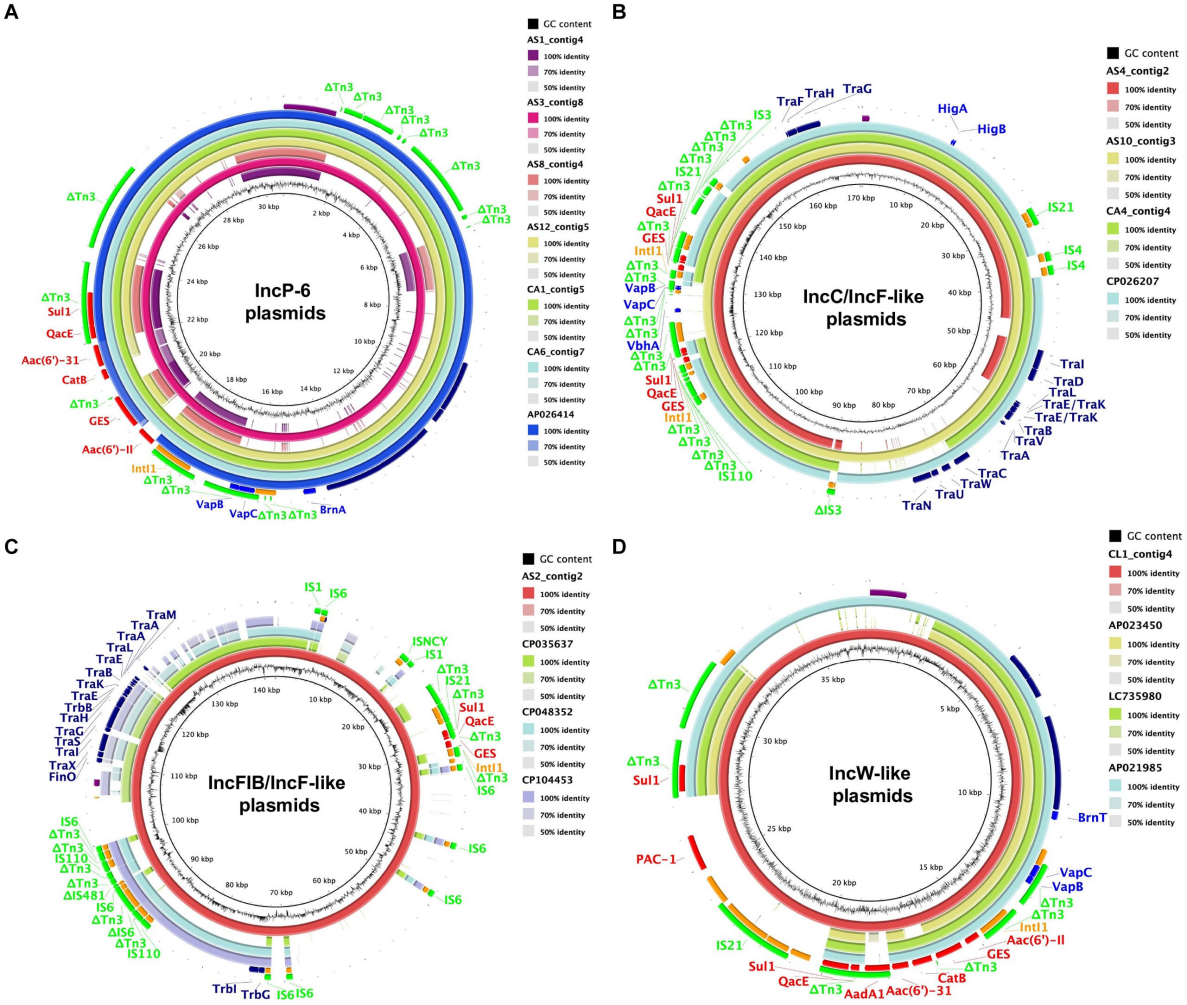
Circular comparison of *bla*_GES_-encoding plasmids with closely related plasmids available in a public database. The reference sequences of AS3_contig8 including IncP-6 **(A)**, AS10_contig3 including IncC/IncF-like **(B)**, AS2_contig2 including IncFIB/IncF-like **(C)**, and CL1_contig4 including IncW-like **(D)** with their shared regions are visualized. Each plasmid is depicted with a ring of different colors and the color intensity shows the nucleotide homologies. The colored arrows represent the positions and directions of specific plasmid modules. Purple, replicon; red, antimicrobial resistance gene; orange, mobile element; light green, IS family; blue, toxin-antitoxin system gene; navy, conjugal transfer gene.

The genetic structure of the *bla*_GES_-encoding plasmids was identified in all GES-type carbapenemase-producing isolates (Figure 1; Table 1). Five of the six *bla*_GES-24_ and *bla*_GES-5_ were localized on IncP-6 plasmids, whereas three of the four *bla*_GES-6_ plasmids were located on IncC plasmids with IncF-like conjugal transfer regions. The remaining *bla*_GES-6_ and *bla*_GES-24_ were localized on IncFIB-containing plasmids with IncF-like regions and a plasmid with an IncW-like replication protein, respectively. These circular closed plasmids ranged in length from 15,748 bp to 170,503 bp. All *bla*_GES_-containing plasmids were located on the class 1 integron cassette of the Tn*3* transposon-related region and contained genes resistant to several antibiotics, including β-lactams, aminoglycosides, quinolones, sulfamides, and chloramphenicol. The *bla*_GES_-containing plasmid of AS10 strain carried two copies of the class 1 integron cassette, each with *bla*_GES-6_. Four of the six IncP-6 plasmids, two of the three IncC/IncF-like plasmids, and one IncW-like plasmid carried a VapBC toxin-antitoxin module. The HigBA toxin-antitoxin system was located on all three IncC/IncF-like plasmids. Partial toxin-antitoxin components, BrnA, VbhA, and BrnT, were detected in the IncP-6, IncC/IncF-like, and IncW-like plasmids, respectively. No toxin-antitoxin region was observed in two IncP-6 and IncFIB/IncF-like plasmids. BLAST analysis of the whole sequence of the IncP-6 plasmid of AS3_contig8 indicated more than 98% identity with the plasmid pKAM644_7 (98% query coverage, AP026414) harbored by environmental *Klebsiella quasipneumoniae* from Japan (Figure 1A). The IncC/IncF-like plasmid AS10_contig3 shared 100% homology and 90% coverage with the *bla*_GES_-noncoding *Escherichia coli* plasmid pECO-dc1b (CP026207) isolated from wastewater and sludge in the United States of America (Figure 1B). The AS2_contig2 plasmid harboring IncFIB/IncF-like regions showed more than 99% identity with *Raoultella ornithinolytica* strain 23 plasmid p23_C (35% query, CP048352) from the environment in Switzerland, *Enterobacter cloacae* strain EN3600 plasmid unnamed5 (34% query, CP035637) from a clinical blood sample in China, and *R. ornithinolytica* strain MQB_Silv_108 plasmid pIncFII_Silv108 (34% query, CP104453) from wastewater in Spain (Figure 1C). The IncW-like CL1_contig4 plasmid displayed similarities to Japanese isolates of *R. ornithinolytica* plasmid pWP3-W18-ESBL-06_2 (AP021985) with 78% coverage and 99% identity from wastewater treatment plant effluent, *Enterobacter roggenkampii* 6 BC1 plasmid p6BC1_GES-24 (LC735980) with 57% coverage and 98% identity from municipal wastewater, and *Enterobacter roggenkampii* OIPH-N260 plasmid pN260-3 (AP023450) with 55% coverage and 99% identity from human bile (Figure 1D).

**Figure 2:**
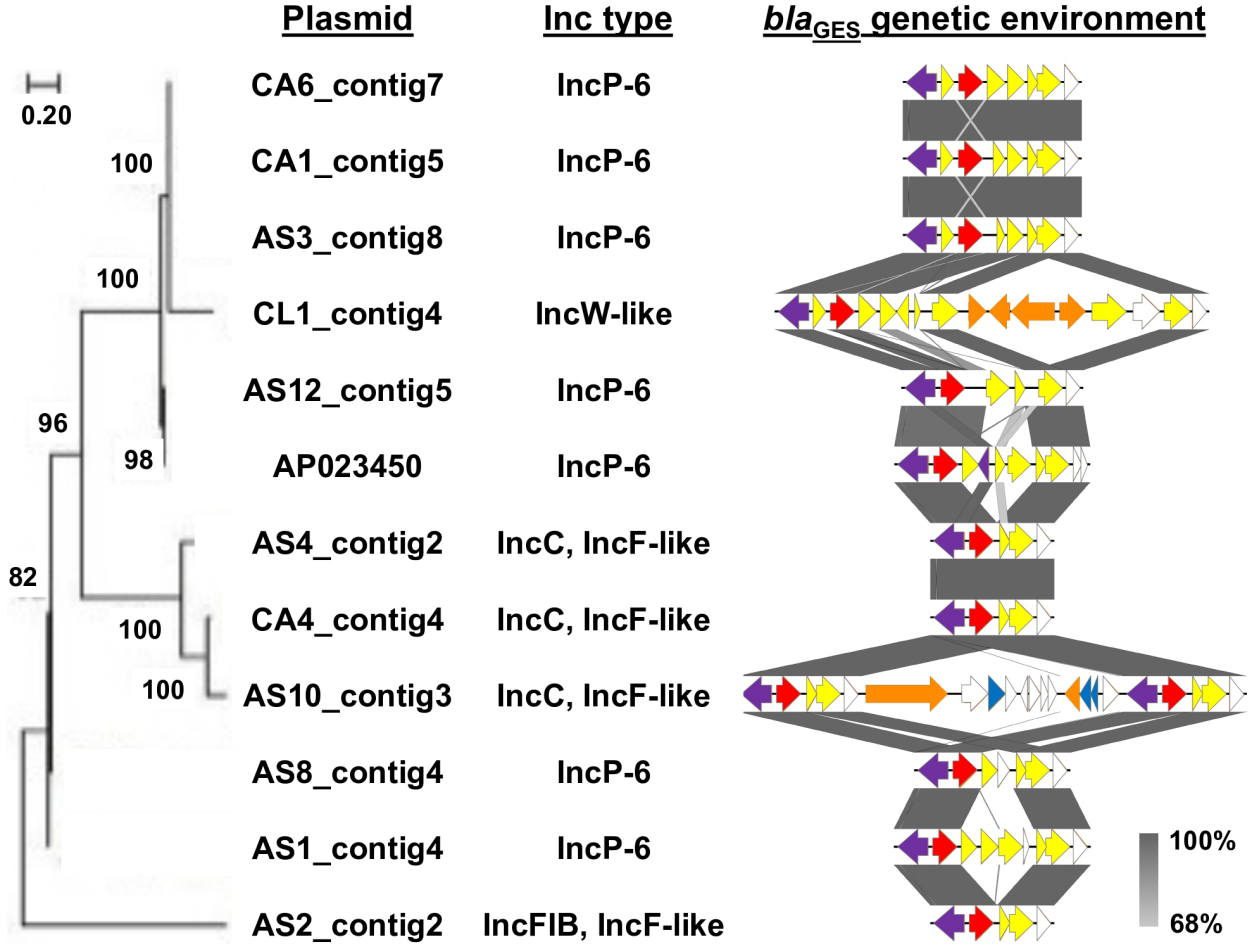
Evolutionary analysis and genetic environment of *bla*_GES_-encoding plasmids. Maximum likelihood phylogenetic tree was generated with a bootstrapping of 1,000 replicates; and bootstrap values are shown on the major nodes. Plasmid names and Inc types are indicated at the right of the tree. Structures of *bla*_GES_ surrounding region into class 1 integron are also represented. The arrows indicate the position and the translation orientation of the coding genes and are colored in accordance with the gene functional classification. The integrase gene, *bla*_GES_, other AMR genes, mobile element, and toxin-antitoxin system gene are highlighted in purple, red, yellow, orange, and blue, respectively. The grey color scale denotes the percentage of sequence identity.

A maximum likelihood phylogenetic tree and the genetic context of the *bla*_GES_-encoding plasmids are shown in Figure 2. Four of the six IncP-6 plasmids showed a closer phylogenetic relationship with the *bla*_IMP-1_- and *bla*_GES-5_-encoding IncP-6 plasmid pN260-3 (AP023450) from Japan. The remaining two IncP-6 plasmids and all IncC/IncF-like plasmids clustered differently. Alignment analysis of the genetic environment of these plasmids showed relatively high background similarity around the *bla*_GES_-containing region. In particular, the *bla*_GES_ genetic structures of the three IncP-6 plasmids (AS3_contig8, CA1_contig5, and CA6_contig7) and IncC/IncF-like plasmids were very similar, whereas the AS10_contig3 plasmid had two structural copies.

### 3.3. Transferability of *bla*_GES_-harboring plasmids

Four isolates with different replicon types of *bla*_GES_-harboring plasmids were evaluated for their ability to transfer *bla*_GES_ to *Escherichia coli* recipient strains. In the conjugation experiment, *bla*_GES_ of AS10 was successfully transferred to the *Escherichia coli* J53 recipient strain (AS10_transconjugant), whereas transconjugants of AS2, AS3, and CL1 were not recovered. The transfer frequency of *bla*_GES_-encoding plasmids in AS10 was 7.8 × 10^−6^. Electrotransformation experiments failed to produce *bla*_GES_-containing transformants from AS2, AS3, and CL1 as confirmed using PCR. We similarly confirmed transferability for isolates with the same IncP-6 plasmid (AS1, AS8, AS12, CA1, and CA6) as AS3, but no transconjugants or transformants were obtained. A scaffold sequence containing *bla*_GES_ was obtained from the assembled short-read data of the AS10_transconjugant. The scaffold data of the AS10_transconjugant showed high homology with AS10_contig3 (Supplementary Figure S1).

### 3.4. Susceptibility profile of GES-type carbapenemase-producing isolates

The antimicrobial susceptibility profiles of the isolates with *bla*_GES_ are described in Table 2. Eight isolates were non-susceptible to carbapenems (imipenem, meropenem, and doripenem), while three were susceptible to them. Regarding non-β-lactam antibiotics, more than half of the isolates were resistant to tobramycin (4/8; 50%), ciprofloxacin (7/11, 64%), and colistin (7/8, 88%). Transconjugants carrying *bla*_GES_ from AS10 were resistant to most β-lactam antibiotics.

**Table 2:** Susceptibility profile of *bla*_GES_-producing isolates, *Escherichia coli* J53 recipient strain, and the transconjugant.

Antibiotics	Minimum inhibitory concentration (μg/mL)												
	AS1	AS2	AS3	AS4	AS8	AS10	AS12	CL1	CA1	CA4	CA6	J53	AS10_transconjugant
Piperacillin	32 (R)	64 (R)	32 (R)	>64 (R)	16 (SDD)	64 (R)	64	8	>64 (R)	>64	4 (S)	≦0.5 (S)	16 (SDD)
Piperacillin/Tazobactam	8/4 (S)	32/4 (R)	16/4 (SDD)	32/4 (R)	≦2/4 (S)	32/4 (R)	8/4 (S)	4/4 (S)	16/4 (SDD)	64/4 (I)	4/4 (S)	≦2/4 (S)	16/4 (SDD)
Cefoperazone/Sulbactam	≦8/8	32/32	16/16	16/16	≦8/8	16/16	16/16	≦8/8	16/16	32/32	16/16	≦8/8	≦8/8
Cefozopran	1	8	>16	16	≦0.5	16	16	4	2	2	4	≦0.5	4
Ceftriaxone	2 (I)	>2 (R)	2 (I)	>2 (R)	>2 (R)	>2 (R)	>2 (R)	>2 (R)	2 (I)	>2 (R)	>2 (R)	≦1 (S)	2 (I)
Ceftazidime	4 (S)	>32 (R)	4 (S)	32 (R)	4 (S)	32 (R)	4 (S)	8 (I)	4 (S)	16 (R)	2 (S)	≦1 (S)	8 (I)
Cefepime	≦0.5 (S)	1 (S)	2 (S)	≦0.5 (S)	≦0.5 (S)	8 (SDD)	4 (I)	≦0.5 (S)	1 (S)	≦0.5 (S)	1 (S)	≦0.5 (S)	≦0.5 (S)
Aztreonam	≦2 (S)	>16 (R)	≦2 (S)	>16 (R)	16 (R)	>16 (R)	8 (I)	≦2 (S)	≦2 (S)	≦2 (S)	≦2 (S)	≦2 (S)	≦2 (S)
Imipenem	4 (R)	8 (R)	>16 (R)	1 (S)	≦0.5 (S)	4 (R)	4 (R)	≦0.5 (S)	4 (R)	2 (I)	4 (R)	≦0.5 (S)	4 (R)
Meropenem	4 (R)	8 (R)	16 (R)	≦0.5 (S)	≦0.5 (S)	4 (R)	4 (R)	≦0.5 (S)	4 (R)	2 (I)	2 (I)	≦0.5 (S)	4 (R)
Doripenem	2 (I)	8 (R)	8 (R)	≦1 (S)	≦1 (S)	8 (R)	4 (R)	≦1 (S)	2 (I)	2 (I)	4 (R)	≦1 (S)	2 (I)
Gentamicin	≦1 (S)	2 (S)	≦1 (S)	4 (S)	≦1 (S)	>8 (R)	8 (I)	2 (S)	≦1 (S)	≦1 (S)	≦1 (S)	≦1 (S)	≦1 (S)
Amikacin	≦4 (S)	≦4 (S)	32 (I)	≦4 (S)	32 (I)	>32 (R)	≦4 (S)	8 (S)	8 (S)	≦4 (S)	16 (S)	≦4 (S)	≦4 (S)
Tobramycin	≦1 (S)	8 (I)	>8 (R)	>8 (R)	>8 (R)	>8 (R)	>8	>8	8 (I)	≦1	8 (I)	≦1 (S)	≦1 (S)
Minocycline	4 (S)	4 (S)	8 (I)	2 (S)	4 (S)	8 (I)	≦1	8	8 (I)	2	8 (I)	≦1 (S)	≦1 (S)
Levofloxacin	2 (R)	1 (I)	4 (R)	>4 (R)	1 (I)	2 (R)	2 (S)	2 (S)	4 (R)	≦0.5 (S)	≦0.5 (S)	≦0.5 (S)	≦0.5 (S)
Ciprofloxacin	2 (R)	1 (R)	>2 (R)	>2 (R)	1 (R)	1 (R)	1 (S)	1 (S)	>2 (R)	≦0.25 (S)	≦0.25 (S)	≦0.25 (S)	≦0.25 (S)
Sulfamethoxazole/Trimethoprim	≦9.5/0.5 (S)	19/1 (S)	≦9.5/0.5 (S)	≦9.5/0.5 (S)	>38/2 (R)	>38/2 (R)	>38/2 (R)	>38/2 (R)	38/2 (S)	>38/2 (R)	19/1 (S)	≦9.5/0.5 (S)	≦9.5/0.5 (S)
Colistin	>4 (R)	>4 (R)	>4 (R)	>4 (R)	>4 (R)	>4 (R)	2	>4	≦1 (I)	>4	>4 (R)	≦1 (I)	≦1 (I)

S, susceptible, I, intermediate, R, resistant, SDD, susceptible-dose dependent.

## 4. Discussion

The incidence of carbapenemase-producing infections significantly increases mortality and medical costs (Gasink et al., 2009; Otter et al., 2017). GES-type carbapenemase producers are present in hospitals for prolonged time, causing outbreaks within hospitals (Boyd et al., 2015; Yamasaki et al., 2017; Ellington et al., 2020; Literacka et al., 2020; Nakanishi et al., 2022; Yoo et al., 2023). However, the genetic features of resistant isolates are not well understood, and their diffusion capabilities could be underestimated. In this study, we characterized diverse plasmids carrying GES-type carbapenemase genes in a sample of hospital sewage in Japan. The existence of a wide variety of GES-type carbapenemase producers within the same sample raises concerns about their potential to promote genetic variability in the environment. Furthermore, these *bla*_GES_-containing plasmids have a novel genetic context with two integron cassettes and several toxin-antitoxin-related regions involved in plasmid maintenance (Yang and Walsh, 2017), indicating their contribution to the evolution and dissemination of resistance. These results have key implications for hygiene because carbapenems are terminal use antibiotics whose resistance may disseminate to the environment through hospital effluents (Mills and Lee, 2019).

Hospital sewage, which is abundant in pathogens and residual antimicrobials, can serve as a persistent reservoir for AMR (Zhang et al., 2020). Antimicrobial regimens are dependent on the patients being treated at each hospital; hospital drug usage influences the bacterial composition and AMR gene abundance in hospital wastewater (Guo et al., 2021; Perry et al., 2021). In routine wastewater-based monitoring, AMR gene profiles are distinct among hospitals using varying amounts of antibiotics, thus highlighting the prevalence of AMR in hospitals (Majlander et al., 2021). Therefore, hospital effluents with selective pressure from environmentally persistent antibiotics can act as major sources of AMR, thereby increasing the risk of AMR dissemination and infection caused by AMR isolates. A study analyzing samples from hospital wastewater treatment plants identified *bla*_GES_ as one of the dominant β-lactamase genes (Hubeny et al., 2021). Indeed, GES-type carbapenemase producers have been identified in hospital wastewater samples from various countries (Gomi et al., 2018; Suzuki Y. et al., 2020; Maehana et al., 2021; Conte et al., 2022; Takizawa et al., 2022; Zagui et al., 2023), which is consistent with our results. Additionally, *bla*_GES_-harboring isolates have been widely detected in wastewater treatment plants (Girlich et al., 2012; Urase et al., 2022), suggesting that the resistance genes from sewage may also be disseminated into communities. Hence, hospital sewage is a potential hazard for transferring resistant organisms to the environment, which is an important public health issue.

GES-type-β-lactamases have been increasingly reported in gram-negative bacteria; *bla*_GES-5_, *bla*_GES-6_, and *bla*_GES-24_ display carbapenemase activities (Naas et al., 2016; Uechi et al., 2018). The MICs to meropenem in these carbapenemase producers vary widely from ≤0.5 to >32 μg/mL, consistent with the low-level resistance to meropenem reported for GES-type carbapenemases (Ellington et al., 2020). In addition to *bla*_GES-24_, *Aeromonas hydrophila* CL1 contains a novel class C β-lactamase, PAC-1 (Bour et al., 2019) derived from *Pseudomonas aeruginosa* in the plasmid but is still sensitive to meropenem. The 11 GES-type bacteria isolated in this study were classified as genetically distinct strains using whole-genome sequencing-based species identification and MLST analysis. Among the identified genera, *Aeromonas* spp. are ubiquitous bacteria primarily found in most water environments and have recently gained attention as an arsenal of AMR genes and mobile genetic elements in natural environments (Usui et al., 2016; Fernandez-Bravo and Figueras, 2020; Gomes et al., 2021; Maehana et al., 2021; Canellas et al., 2023). Previous studies have reported a higher prevalence of diverse AMR genes and integron cassettes in *Aeromonas* spp. isolates (Fernandez-Bravo and Figueras, 2020; Canellas et al., 2023). Maehana et al. isolated *Aeromonas* spp. carrying four tandem copies of *bla*_GES-24_ from sewage water at a medical institution (Maehana et al., 2021). These observations, coupled with our results, suggest that *Aeromonas* spp. could be a possible bacterial indicator for predicting the dissemination of AMR in hospital sewage.

PlasmidFinder and BLAST analyzes showed that the *bla*_GES_-containing plasmids can be classified into the following four groups: IncP-6, IncC/IncF-like, IncFIB/IncF-like, and IncW-like. While these various types of GES producers have been isolated from patients worldwide (Literacka et al., 2020; Umeda et al., 2021), similar *bla*_GES_-containing plasmids were not obtained from clinical isolates in our hospital. Although phylogenetic tree analysis revealed genetic relatedness in some IncP-6 or IncC plasmids in this study, determining high-resolution plasmid relationships using phylogenetic analysis is still challenging (Orlek et al., 2017; Suzuki M. et al., 2020). In the circular comparison of *bla*_GES_-encoding plasmids, the whole sequences of the IncP-6 and IncW-like plasmids showed high homology with those of *bla*_GES_-encoding plasmids isolated in Japan (Umeda et al., 2021). *bla*_GES_ with the IncP-6 plasmid backbone has also been isolated from both clinical and environmental samples in Poland (Literacka et al., 2020) and Brazil (Conte et al., 2022), suggesting that the plasmid may play an important role in the global dissemination of these genes. BLAST analysis revealed that the full sequences of the IncC/IncF-like and IncFIB/IncF-like plasmids were highly similar to plasmids discovered in various bacterial species in both clinical and environmental samples. The IncF-like conjugative transfer proteins in these plasmids are involved in horizontal gene transfer and may expand their ability to disseminate AMR genes (Lang and Zechner, 2012; Rozwandowicz et al., 2018). The plasmids encoding *bla*_GES_ containing IncC (Takashima et al., 2022) and IncFIB (Teixeira et al., 2022) have been reported globally. We simultaneously detected four plasmid types related to sporadic isolates worldwide in wastewater from a single hospital. Notably, the AS3, CA1, and CA4 strains had nearly identical *bla*_GES_-harboring plasmids, despite belonging to different bacterial species. Thus, *bla*_GES_ may spread by coordinating changes in the diverse genetic structures of plasmids for long-term persistence and could be a major cause of hospital wastewater contamination.

The *bla*_GES_ gene of all plasmids was located on the class 1 integron cassette, along with a Tn*3* transposon-derived sequence. The plasmid backbone was deposited in various bacterial species databases (Umeda et al., 2021). Comparison of the context around *bla*_GES_ revealed that the genetic environment was relatively conserved among the plasmid incompatibility groups. However, AS10_contig3 harbored two class 1 integron cassettes with *bla*_GES_. Tandem copies of *bla*_GES_ exist within an integron cassette (Xu et al., 2018; Maehana et al., 2021; Takizawa et al., 2022); however, multiple copies of the integron cassette with *bla*_GES_ have not been observed. The expression level of AMR genes within integron cassettes was the highest when the gene was in the first cassette (Collis and Hall, 1995; Souque et al., 2021), indicating that the novel *bla*_GES_-surrounding region of AS10_contig3 could potentially increase the total amount of integron cassette expression and phenotypic resistance.

The horizontal transmission of plasmids is critical in the spread of AMR genes. Our conjugation experiment showed that the IncC/IncF-like plasmid was successfully transferred to the recipient strain, whereas the IncP-6, IncFIB/IncF-like, and IncW-like plasmids were not transferred to *Escherichia coli* J53 and C600 recipient strains. The different profiles of conjugal transfer genes in each plasmid may affect their transferability (Zatyka and Thomas, 1998). The transconjugant of *bla*_GES-6_-containing IncC/IncF-like plasmid acquired resistance to most β-lactam antibiotics, including carbapenems, indicating that *bla*_GES-6_ was functional in the *Escherichia coli* parent strain. None of the transformants were obtained from IncP-6, IncFIB/IncF-like, or IncW-like plasmids via electroporation using the *Escherichia coli* HST08 strain. These failures in both conjugation and transformation are consistent with previous findings and may be due to the exclusion of *Escherichia coli* from the host replication range in *bla*_GES_-containing plasmids (Bonnin et al., 2011; Chudejova et al., 2018; Conte et al., 2022). Moreover, the transfer mechanisms responsible for AMR gene acquisition include integron-mediated mobilization of gene cassettes (Fluit and Schmitz, 1999). Thus, our results indicate that integrons, instead of plasmids, may mainly be responsible for the dissemination of *bla*_GES_ among bacteria.

Toxin-antitoxin systems are distributed in environmental plasmids with AMR genes and are known to allow stable plasmid persistence in bacteria (Lee et al., 2015; An et al., 2018; Takashima et al., 2022). Wozniak et al. reported that these systems advance the maintenance of a conjugative element (Wozniak and Waldor, 2009). We showed that most *bla*_GES_-encoding plasmids contained multiple toxin-antitoxin systems and their components. Seven of the 11 plasmids carried the VapBC toxin-antitoxin system, which is associated with growth inhibition to resist environmental stress (Eroshenko et al., 2020). The IncC/IncF-like plasmid, which was successfully conjugated, notably encoded the HigBA toxin-antitoxin system and demonstrated plasmid stability in the environment (Qi et al., 2021).

In summary, we provided insights into the genomic structures of plasmids harboring *bla*_GES_ from different bacterial species in hospital sewage. Our results indicate considerable genetic diversity of *bla*_GES_-encoding plasmids in the same water sample, suggesting their high adaptability to aquatic environments. These findings revealed the importance of healthcare wastewater as a potential reservoir of *bla*_GES_, highlighting the need for serial monitoring of environmentally emerging GES-type carbapenemase producers.

## Data availability statement

Original datasets are available in a publicly accessible repository: The original contributions presented in the study are publicly available. This data can be found in the NCBI database under accession number(s): SAMN33317300 (AS1), SAMN33317301 (AS2), SAMN33317302 (AS3), SAMN33317303 (AS4), SAMN33317304 (AS8), SAMN33317305 (AS10), SAMN33317306 (AS12), SAMN33317307 (CL1), SAMN33317308 (CA1), SAMN33317309 (CA4), and SAMN33317310 (CA6). Further queries can be directed to the corresponding authors.

## Author contributions

RS designed, organized, and coordinated this project. YO was the chief investigator and was responsible for data analysis. IP, SM, YG, YN, and RK contributed to the data interpretation. YO and RS wrote the initial and final drafts of the manuscript. All authors revised the drafts of the manuscript and approved the final version of the manuscript.

## Funding

This work was supported by the Japan Agency for Medical Research and Development (AMED) under Grant Numbers JP20wm0125007 (RS), JP20wm0225013 (RS), JP20wm0225004 (RS), and JP22wm0225022 (RS) and the Japan Society for the Promotion of Science (JSPS) KAKENHI under Grant Number JP23H03551 (YO and RS). These funders had no role in the study design, data collection, analysis, decision to publish, or preparation of the manuscript.

## Conflict of interest

The authors declare that the research was conducted in the absence of any commercial or financial relationships that could be construed as a potential conflict of interest.

## Publisher’s note

All claims expressed in this article are solely those of the authors and do not necessarily represent those of their affiliated organizations, or those of the publisher, the editors and the reviewers. Any product that may be evaluated in this article, or claim that may be made by its manufacturer, is not guaranteed or endorsed by the publisher.

## Footnotes

^1^http://rdp.cme.msu.edu/

^2^http://genomicepidemiology.org/

^3^http://pubmlst.org/

^4^https://mlstkv.insp.mx/

## Abbreviations

AMR: antimicrobial resistance
BLAST: Basic Local Alignment Search Tool
BTB: bromothymol blue
GES: Guiana extended-spectrum
Inc.: incompatibility
LB: Luria-Bertani
MIC: minimum inhibitory concentrations
MLST: multi-locus sequence typing
PCR: polymerase chain reaction
ST: sequence type
