# Supplementary material for: Novel insights into genetic characteristics of blaGES-encoding plasmids from hospital sewage
Source: Front Microbiol. 2023 Aug 17;14:1209195. doi: 10.3389/fmicb.2023.1209195 (PMC10469963; doi:10.3389/fmicb.2023.1209195)
Supplement: Supplementary file 1 [file Table_1.DOCX]

**Supplementary Table 1. Strain information isolated from hospital sewage.**

| Strain | 16S rRNA gene sequencing | DHL agar | Carbapenemase-selective agar | Carbapenemase genes |
| --- | --- | --- | --- | --- |
| BTB1 | *Klebsiella aerogenes* | + | - | Not determined |
| BTB2 | *Escherichia coli* | + | - | Not determined |
| BTB4 | *Klebsiella quasipneumoniae* | + | - | Not determined |
| BTB5 | *Raoultella ornithinolytica* | + | - | Not determined |
| BTB6 | *Enterobacter bugandensis* | + | - | Not determined |
| BTB7 | *Pseudomonas aeruginosa* | + | + | - |
| BTB8 | *Klbsiella oxytoca* | + | - | Not determined |
| BTB9 | *Klebsiella pneumoniae* | + | - | Not determined |
| BTB10 | *Acinetobacter gyllenbergii* | + | + | - |
| BTB11 | *Aeromonas caviae* | + | - | Not determined |
| BTB12 | *Kosakonia oryzae* | + | - | Not determined |
| BTB13 | *Klebsiella variicola* | + | - | Not determined |
| BTB16 | *Shewanella seohaensis* | + | - | Not determined |
| AS1 | *Enterobacter soli* | + | + | GES |
| AS2 | *Enterobacter ludwigii* | + | + | GES |
| AS3 | *Klebsiella variicola* | + | + | GES |
| AS4 | *Enterobacter tabaci* | + | + | GES |
| AS5 | *Burkholderia cepacia* | + | + | - |
| AS6 | *Enterobacter tabaci* | + | + | - |
| AS8 | *Enterobacter ludwigii* | + | + | GES |
| AS10 | *Klebsiella variicola* | + | + | GES |
| AS12 | *Aeromonas hydrophila* | + | + | GES |
| AS13 | *Enterobacter tabaci* | + | + | - |
| AS14 | *Aeromonas caviae* | + | + | - |
| CL1 | *Aeromonas hydrophila* | + | + | GES |
| CL4 | *Pseudomonas aeruginosa* | + | + | - |
| CL5 | *Serratia marcescens* | + | - | Not determined |
| CL8 | *Raoultella electrica* | + | - | Not determined |
| CA1 | *Klebsiella quasipneumoniae* | + | + | GES |
| CA2 | *Aeromonas caviae* | + | + | - |
| CA3 | *Pseudomonas mosselii* | + | + | - |
| CA4 | *Aeromonas taiwanensis*/*dhakensis* | + | + | GES |
| CA5 | *Aeromonas hydrophila* | + | + | - |
| CA6 | *Serratia marcescens* | + | + | GES |
| CA8 | *Pseudomonas taiwanensis* | + | + | - |
| CA9 | *Stenotrophomonas maltophilia* | + | + | - |
| CA12 | *Enterobacter tabaci* | + | + | - |
| CA13 | *Pseudomonas otitidis* | + | + | - |
