# Supplementary figures and images for: Novel insights into genetic characteristics of blaGES-encoding plasmids from hospital sewage
Source: Front Microbiol. 2023 Aug 17;14:1209195. doi: 10.3389/fmicb.2023.1209195 (PMC10469963; doi:10.3389/fmicb.2023.1209195)

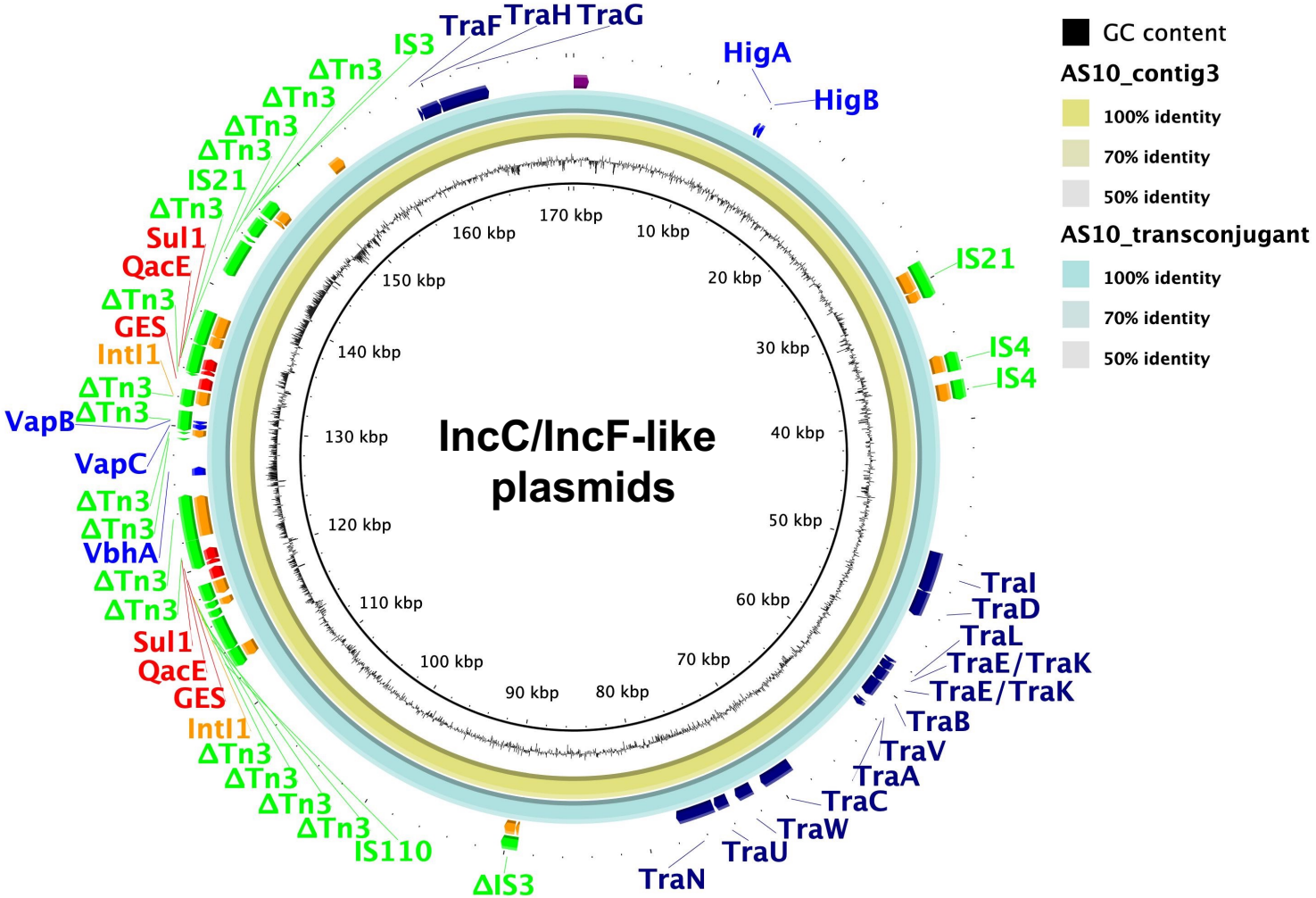

Supplement: Supplementary file 2 [file Image_1.pdf]
